# Supplementary material for: Desmoplastic Small Round Cell Tumor of the Uterus: A Report of Molecularly Confirmed Case with EWSR1-WT1 Fusion
Source: Diagnostics (Basel). 2022 May 10;12(5):1184. doi: 10.3390/diagnostics12051184 (PMC9140206; doi:10.3390/diagnostics12051184)
Supplement: Supplementary file 1 [file diagnostics-12-01184-s001.zip › diagnostics-1679856-supplementary.pdf]

Supplementary Table S1. List of immunohistochemical antibodies

Antibody	Clone	Dilution	Producer	Platform	Detection
transgelin	2A10C2	1:300	Cell Marque, Rocklin, CA, USA	Ventana BenchMark ULTRA (Roche, Basel, Switzerland)	OptiView
CD56	123C3.D5	1:25	Zeta Corporation, Sierra Madre, CA, USA	PT-link (Agilent, Santa Clara, CA, USA)	EnVision FLEX (Agilent)
CD57	TB01	1:100	Dako, Glostrup, Denmark	PT-link (Agilent, Santa Clara, CA, USA)	EnVision FLEX (Agilent)
EMA	E29	1:200	Dako, Glostrup, Denmark	PT-link (Agilent, Santa Clara, CA, USA)	EnVision FLEX (Agilent)
cyclin D1	EP12	RTU	Dako, Glostrup, Denmark	Ventana BenchMark ULTRA (Roche, Basel, Switzerland)	OptiView
CD10	56C6	1:50	Novocastra, Leica Biosystems, Wetzlar, Germany	Ventana BenchMark ULTRA (Roche, Basel, Switzerland)	OptiView
desmin	D33	1:200	Dako, Glostrup, Denmark	Ventana BenchMark ULTRA (Roche, Basel, Switzerland)	OptiView
h-caldesmon	h-CD	1:50	Novocastra, Leica Biosystems, Wetzlar, Germany	PT-link (Agilent, Santa Clara, CA, USA)	EnVision FLEX (Agilent)
cytokeratin	AE1/AE3	1:200	Dako, Glostrup, Denmark	Dako Omnis, Agilent, Santa Clara, CA, USA	EnVision FLEX (Agilent)
BerEP4	BerEP4	1:100	Dako, Glostrup, Denmark	PT-link (Agilent, Santa Clara, CA, USA)	EnVision FLEX (Agilent)
NSE	BBS-NC-VI-H14	1:400	Dako, Glostrup, Denmark	PT-link (Agilent, Santa Clara, CA, USA)	EnVision FLEX (Agilent)
IFITM1	-	1:300	Abcam, Cambridge, United Kingdom	PT-link (Agilent, Santa Clara, CA, USA)	EnVision FLEX (Agilent)

WT1	6F-H2	1:100	Dako, Glostrup, Denmark	PT-link (Agilent, Santa Clara, CA, USA)	EnVision FLEX (Agilent)
p53	BP 53-12	1:200	Zytomed Systems, Berlin, Germany	PT-link (Agilent, Santa Clara, CA, USA)	EnVision FLEX (Agilent)
Ki-67	MIB1	1:100	Dako, Glostrup, Denmark	Ventana BenchMark ULTRA (Roche, Basel, Switzerland)	UltraView
myogenin	FD5	1:50	Dako, Glostrup, Denmark	PT-link (Agilent, Santa Clara, CA, USA)	EnVision FLEX (Agilent)
synaptophysin	DAK-SYNAP	1:100	Dako, Glostrup, Denmark	Dako Omnis, Agilent, Santa Clara, CA, USA	EnVision FLEX (Agilent)
chromogranin	LK2H10	1:400	Zytomed Systems, Berlin, Germany	PT-link (Agilent, Santa Clara, CA, USA)	EnVision FLEX (Agilent)
PAX8	-	1:50	Cell Marque, Rocklin, CA, USA	Dako Omnis, Agilent, Santa Clara, CA, USA	EnVision FLEX (Agilent)
CD117	-	1:200	Dako, Glostrup, Denmark	Ventana BenchMark ULTRA (Roche, Basel, Switzerland)	UltraView
